# Supplementary material for: Equation-of-Motion Coupled-Cluster Variants in Combination with Perturbative Triples Corrections in Strong Magnetic Fields
Source: J Chem Theory Comput. 2025 Oct 6;21(20):10177–92. doi: 10.1021/acs.jctc.5c00779 (PMC12573758; doi:10.1021/acs.jctc.5c00779)
Supplement: Supplementary file 1 [file ct5c00779_si_001.pdf]

Supporting information for Equation-of-motion coupled-cluster variants in combination with perturbative triples corrections in strong magnetic fields

Marios-Petros Kitsaras[1,2,3], Florian Hampe[3,4], Lena Reimund[3], Stella Stopkowicz[2,3,4,5]

1 Laboratoire de Chimie et Physique Quantiques - UMR5626, CNRS, Université de Toulouse - Bat. 3R1b4 - 118 route de Narbonne, F-31062, Toulouse, France
2 Fachrichtung Chemie, Universität des Saarlandes, D-66123 Saarbrücken, Germany
3 Department Chemie, Johannes Gutenberg-Universität Mainz, Duesbergweg 10-14, D-55128 Mainz, Germany
4 Centre for Advanced Study (CAS) at the Norwegian Academy of Science and Letters, Drammensveien 78, N-0271 Oslo, Norway
5 Hylleraas Centre for Quantum Molecular Sciences, Department of Chemistry, University of Oslo, P.O. Box 1033 Blindern, N-0315 Oslo, Norway

I. Li atom in strong magnetic field

The electron affinity of lithium ($1^2S_g/1^2\Sigma_g \rightarrow 1^1S_g/1^1\Sigma_g$) is determined in a magnetic field between 0-0.5 B_0. Tab. S1 shows the energy differences between the total energies of the lithium atom in the $1^2S_g/1^2\Sigma_g$ state and the lithium anion in the $1^1S_g/1^1\Sigma_g$ state. The corresponding calculations have been performed using a Cartesian (cart) uncontracted (unc) aug-cc-pCVQZ basis set. The field-free electron affinity is in excellent agreement with the Ref.[1] in which extensive ANO basis sets were employed and the IP-EOM and EA-EOM results are essentially identical. The difference between the approaches amounts to 0.021 eV and 0.002 eV, respectively. These deviations hardly change in a magnetic field with a maximum of 0.030 eV for IP-EOM and 0.007 eV for EA-EOM at $B = 0.5$ B_0.

II. A comparison between Koopman's theorem and IP/EA-EOM-CCSD

In this section, the lowest IPs of C and the EAs of F using Koopman's approach and at the IP/EA-EOM-CCSD level of theory are presented without considering the energy of the free electron. As such, the former exactly correspond to the energy difference between the ground state of the neutral system and the lowest states of the cation and anion, respectively. These plots serve first to underline the importance of correlation in such predictions and second to provide a reference to understand the effects of the Landau energy of the free electron to the IP and EA paths.

For the carbon atom with the $^3P_g/^3\Pi_g^-$ ground state, the resulting IP-EOM energies are plotted as a function of the magnetic-field strength in Fig. S1. Up to $B = 0.191$ B_0, the IP-EOM energy is rising as the electron to be removed comes from the $2p_0^\beta$ orbital which gets stabilized in the field due to the spin-Zeeman term. For higher field strengths, ionization occurs from the $2s^\alpha$ orbital which in contrast is energetically destabilized. The corresponding IPs using Koopman's theorem already cross for $B \approx 0.06$ B_0 which, however, does not entirely coincide with the respective crossing for the IP-EOM energy. This means, that Koopmans' picture is off by more than 30785T and should not be consulted for systems in finite magnetic fields.

Fig. S2 shows the lowest EA-EOM energies obtained for the fluorine atom in the $^2P_u/^2\Pi_u^-$ ground state up to a field strength of $B = 0.25$ B_0 compared to predictions using Koopman's theorem. The EA-EOM energy for the attachment of an α electron in the $2p_{+1}$ orbital is increasing steeply up to

Table S1: Differences between the total energies of the lithium atom in the $1^2S_g/1^2\Sigma_g$ state and the lithium anion in the $1^1S_g/1^1\Sigma_g$ state given in eV in a magnetic field between 0-0.5 B_0 obtained via ΔCCSD, IP-EOM-CCSD, and EA-EOM-CCSD.

B / B_0	$\Delta E_{\Delta\text{CCSD}}$	$\Delta E_{\text{IP-EOM-CCSD}}$	$\Delta E_{\text{EA-EOM-CCSD}}$
0.00	−0.608	−0.629	−0.606
0.05	0.325	0.305	0.324
0.10	1.532	1.513	1.529
0.15	2.851	2.832	2.848
0.20	4.222	4.202	4.217
0.25	5.612	5.591	5.607
0.30	7.004	6.981	6.998
0.35	8.390	8.365	8.383
0.40	9.766	9.740	9.760
0.45	11.135	11.106	11.128
0.50	12.497	12.467	12.490

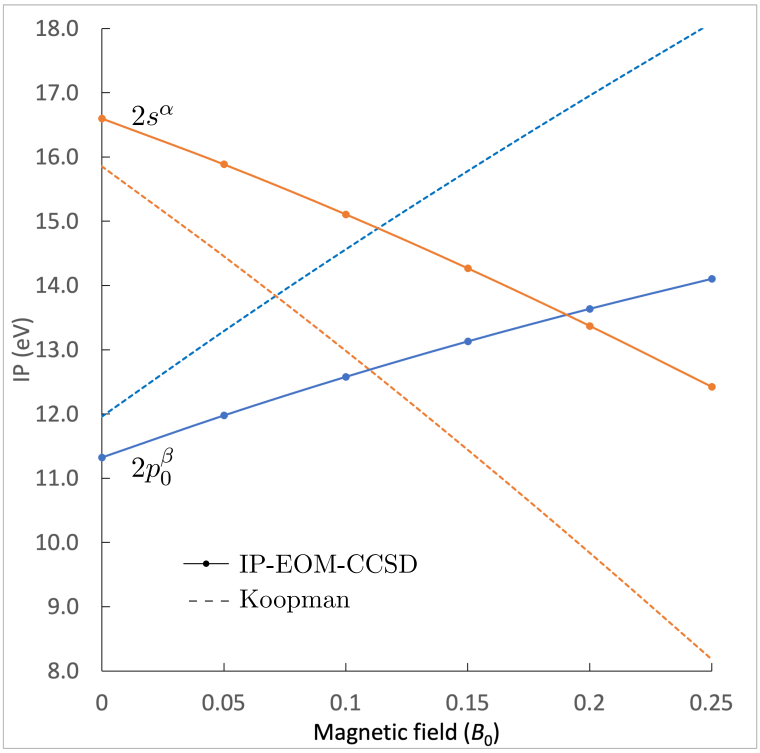

Figure S1: Lowest IP-EOM energy for the carbon atom in the $^3\text{P}_g/^3\Pi_g^-$ state in a magnetic field between 0-0.25 B_0 involving ionization from the $2p_0^\beta$ and the $2s^\alpha$ spin orbital, respectively at the EOM-CCSD and Koopman's levels of theory without the Landau correction.

$B \approx 0.13$ B_0 implying that adding an electron to fluorine to yield fluoride is harder in a magnetic field. For stronger fields, the increase flattens as for these magnetic-field strengths a β electron is added in the $3p_{-1}$ orbital. For even stronger fields ($B \gtrapprox 0.2$ B_0) the preferred additional electron is a β electron to the $3d_{-2}$ orbital. The resulting anionic state is stabilized with increasing magnetic-field strength. While the second change almost coincides with the crossing of the orbital energies of the $3p_{-1}$ and $3d_{-2}$ orbital, the first occurs for much higher field strengths than the corresponding orbital energies suggest. This can be understood in the following way: By adding a $2p_{+1}$ electron, a stable noble gas configuration is obtained. The stabilization due to the noble-gas electron configuration is also seen by the big deviation of the Koopman's predictions relative to the EA-EOM-CCSD results for the blue line. This process thus is preferred over adding a $3p_{-1}$ electron even for field strengths for which the

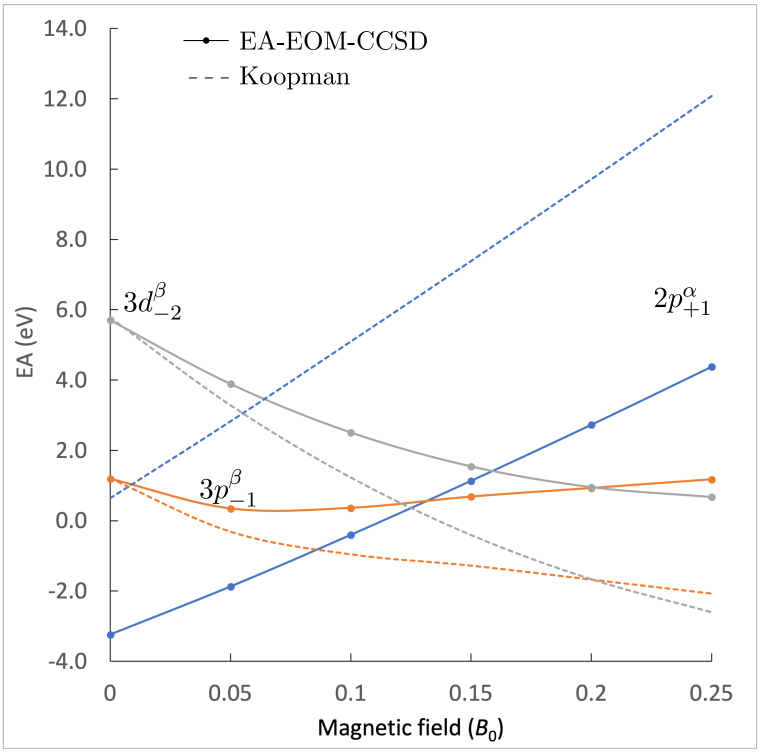

Figure S2: Lowest EA-EOM energies for the fluorine atom in the $^2\mathrm{P}_u/^2\Pi_u^-$ state in a magnetic field between 0-0.25 B_0 involving electron attachment in the $2p^{\alpha}_{+1}$, $3p^{\beta}_{-1}$, and the $3d^{\beta}_{-2}$ spin orbital, respectivelyat the EA-EOM-CCSD and Koopman's level of theory without the Landau correction.

energy of the $3p_{-1}$ orbital is already lower than the $2p_{+1}$ orbital. As shown for ionization energies, Koopmans' picture is off by more than 29000T, due to correlation and relaxation effects.

III. Ionisation potentials and electron affinities

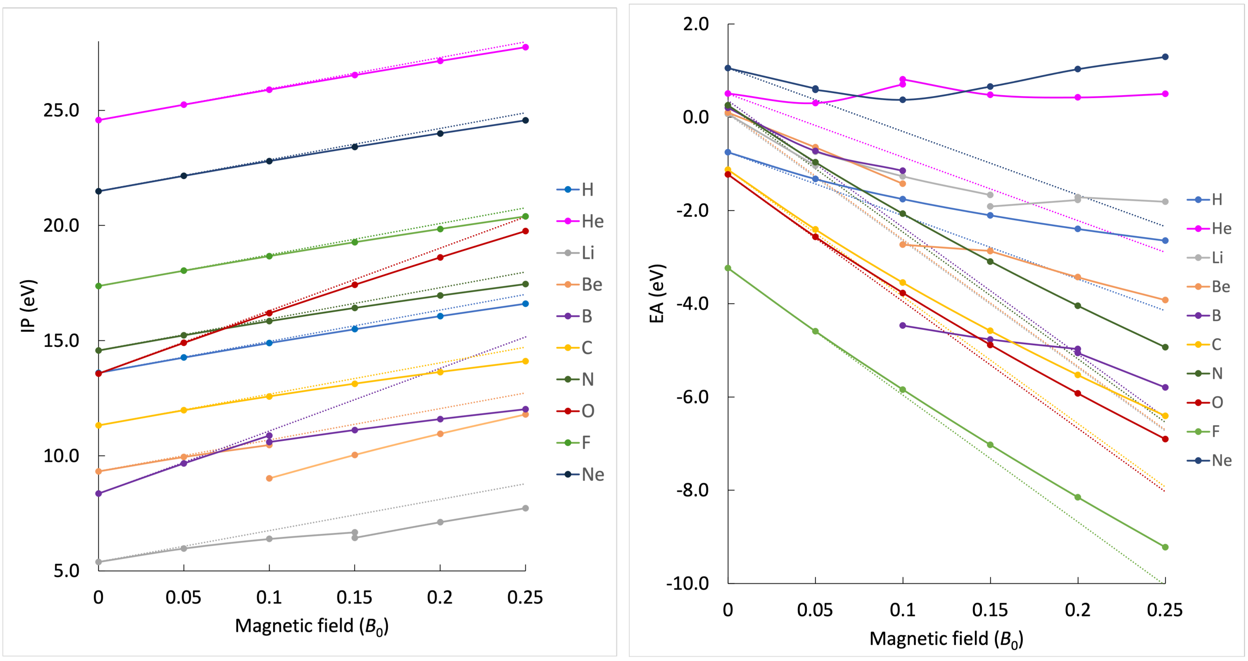

(a) Ionisation potentials

(b) Electron affinities

Figure S3: Landau-corrected IPs(a) and EAs(b) of the atoms H-Ne in a magnetic field between 0-0.25 B_0 at the EA-EOM-CCSD level of theory. The finite-field results are compared against a simple perturbative consideration (dotted) which only consist of the diamagnetic Landau contribution.

The IPs and EAs from the finite-magnetic field calculations are plotted as a function of the magnetic field together with a simple perturbative consideration as a dotted line in Fig. S3. In fact the perturbative consideration only consists of the diamagnetic Landau contribution

$$\frac{1}{2}\left(1+|m_l|\right)\cdot B.$$

This is added in the case of IPs or subtracted in the case EAs. The only factor that contributes is the angular momentum projection of the leaving or attached electron m_l. This contribution defines the slope of the curves in weak fields. In the plots, the shift from a linear behaviour of the finite-field predictions can be clearly observed.

IV. Triples contributions at the EOM-CCSD(T)(a)* level of theory for the IPs of Na and Mg

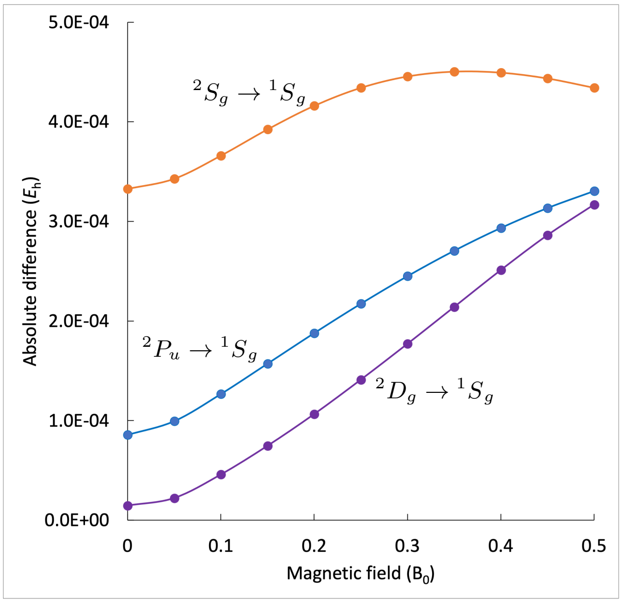

(a) Na

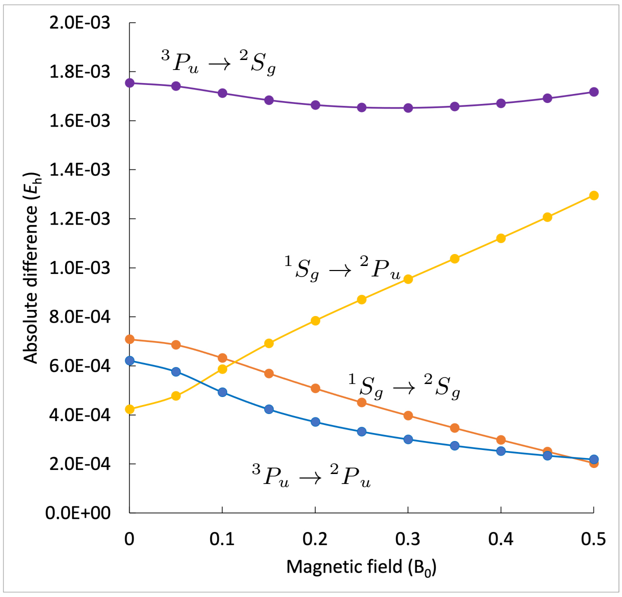

(b) Mg

Figure S4: The absolute difference between the IPs calculated at the CCSD and CCSD(T)(a)* levels of theory for Na (a) and Mg (b).

The triples contributions at the EOM-CCSD(T)(a)* for the IPs of the Na and Mg atoms are estimated by calculating the absolute differences between the predictions at the CCSD and CCSD(T)(a)* levels of theory. They are plotted in Fig. S4 as a function of the magnetic-field strength. Given the scale of the plots it is safe to assume that the error is relatively constant in different magnetic-field strengths with no recognisable patterns. The difference remains below 1 mE_h for Na. For Mg, the triples contributions for the $^3P_u \to {}^2S_g$ transition are calculated consistently around 1.8 mE_h, while they exceed 1 mE_h for the $^1S_g \to {}^2P_u$ transition for a magnetic field stronger than 0.25 B_0. The $^1S_g \to {}^2S_g$ and $^3P_u \to {}^2P_u$ transition exhibit triples contributions below 1 mE_h with a decreasing tendency when increasing the magnetic-field strength.

Table S2: EOM-EA-CCSD transition dipole moments $|\mu_{i\to f}|^2$ in $e^2a_0^2$ for selected electronic transitions between low-lying doublet states of the sodium atom in a magnetic field between 0-0.5 B_0. Differences $\Delta_{\mathrm{EA-EE}}$ to results obtained by EOM-EE-CCSD calculations are listed.

B / B_0	$\lvert\mu_{1^2\Sigma_g\to 1^2\Pi_{\pm1,u}}\rvert^2$	$\Delta_{\mathrm{EA-EE}}$	$\lvert\mu_{1^2\Sigma_g\to 1^2\Sigma_u}\rvert^2$	$\Delta_{\mathrm{EA-EE}}$
0.00	6.299047	0.002597	6.299047	0.002597
0.02	6.241167	0.002505	6.296609	0.002576
0.04	6.072672	0.002301	6.288928	0.002531
0.06	5.822137	0.002078	6.276363	0.002485
0.08	5.529566	0.001874	6.261231	0.002455
0.10	5.225684	0.001698	6.247059	0.002443
0.12	4.928337	0.001544	6.237233	0.002444
0.14	4.646086	0.001406	6.234145	0.002453
0.16	4.382048	0.001278	6.239104	0.002464
0.18	4.136534	0.001159	6.252629	0.002471
0.20	3.908602	0.001044	6.274754	0.002468
0.22	3.696881	0.000935	6.305214	0.002584
0.24	3.499958	0.000830	6.343517	0.002419
0.26	3.316532	0.000729	6.388961	0.002370
0.28	3.145450	0.000633	6.440636	0.002301
0.30	2.985696	0.000541	6.497446	0.002214
0.32	2.836368	0.000454	6.558141	0.002108
0.34	2.696653	0.000373	6.621375	0.001985
0.36	2.565807	0.000297	6.685768	0.001847
0.38	2.443152	0.000226	6.749966	0.001697
0.40	2.328062	0.000159	6.812695	0.001535
0.42	2.219965	0.000098	6.872807	0.001365
0.44	2.118338	0.000042	6.929303	0.001190
0.46	2.022704	-0.000009	6.981351	0.001013
0.48	1.932630	-0.000057	7.028287	0.000841
0.50	1.847725	-0.000099	7.069611	0.000665

V. Transition dipole moments for the Na atom

In ref. [2], we have investigated selected transitions of the sodium atom together with their corresponding squared transition dipole moments (STMs) calculated using the finite-field EOM-EE-CCSD approach. Here, we report corresponding STMs for s→p transitions calculated at the EOM-EA-CCSD level starting from a Na^+ reference together with the differences (see table S2) to the values reported in ref. [2]. Evidently, the STMs are very similiar with differences of at most the third decimal place. Hence, for the prediction of intensities, EOM-EA-CCSD transition dipole moments can readily be used alternatively to EOM-EE-CCSD when desired.

References

[1] M. Nooijen and R. J. Bartlett, “Equation of motion coupled cluster method for electron attachment”, J. Chem. Phys. **102**, 3629–3647 (1995).

[2] F. Hampe and S. Stopkowicz, “Transition-dipole moments for electronic excitations in strong magnetic fields using equation-of-motion and linear response coupled-cluster theory”, J. Chem. Theory Comput. **15**, 4036–4043 (2019).
